# Particulate Matter Exposure Exacerbates High Glucose-Induced Cardiomyocyte Dysfunction through ROS Generation

**DOI:** 10.1371/journal.pone.0023116

**Published:** 2011-08-05

**Authors:** Li Zuo, Dane J. Youtz, Loren E. Wold

**Affiliations:** 1 Center for Cardiovascular and Pulmonary Research, The Research Institute at Nationwide Children's Hospital, Columbus, Ohio, United States of America; 2 Department of Pediatrics, The Ohio State University College of Medicine, Columbus, Ohio, United States of America; 3 Department of Physiology and Cell Biology, The Ohio State University College of Medicine, Columbus, Ohio, United States of America; Roswell Park Cancer Institute, United States of America

## Abstract

Diabetes mellitus and fine particulate matter from diesel exhaust (DEP) are both important contributors to the development of cardiovascular disease (CVD). Diabetes mellitus is a progressive disease with a high mortality rate in patients suffering from CVD, resulting in diabetic cardiomyopathy. Elevated DEP levels in the air are attributed to the development of various CVDs, presumably since fine DEP (<2.5 µm in diameter) can be inhaled and gain access to the circulatory system. However, mechanisms defining how DEP affects diabetic or control cardiomyocyte function remain poorly understood. The purpose of the present study was to evaluate cardiomyocyte function and reactive oxygen species (ROS) generation in isolated rat ventricular myocytes exposed overnight to fine DEP (0.1 µg/ml), and/or high glucose (HG, 25.5 mM). Our hypothesis was that DEP exposure exacerbates contractile dysfunction via ROS generation in cardiomyocytes exposed to HG. Ventricular myocytes were isolated from male adult Sprague-Dawley rats cultured overnight and sarcomeric contractile properties were evaluated, including: peak shortening normalized to baseline (PS), time-to-90% shortening (TPS_90_), time-to-90% relengthening (TR_90_) and maximal velocities of shortening/relengthening (±dL/dt), using an IonOptix field-stimulator system. ROS generation was determined using hydroethidine/ethidium confocal microscopy. We found that DEP exposure significantly increased TR_90_, decreased PS and ±dL/dt, and enhanced intracellular ROS generation in myocytes exposed to HG. Further studies indicated that co-culture with antioxidants (0.25 mM Tiron and 0.5 mM N-Acetyl-L-cysteine) completely restored contractile function in DEP, HG and HG+DEP-treated myocytes. ROS generation was blocked in HG-treated cells with mitochondrial inhibition, while ROS generation was blocked in DEP-treated cells with NADPH oxidase inhibition. Our results suggest that DEP exacerbates myocardial dysfunction in isolated cardiomyocytes exposed to HG-containing media, which is potentially mediated by various ROS generation pathways.

## Introduction

Diabetes mellitus is recognized as one of the key risk factors in the development of cardiovascular disease (CVD) [1]. Air pollution exposure has been shown to directly relate to an increase in cardiopulmonary deaths in highly polluted urban settings [2], [3]. It has also been recognized that diabetics are more susceptible to air pollution-induced heart dysfunction, although the underlying mechanisms remain understood [1], [4], [5], [6], [7]. Fine particulate matter (PM; <2.5 µm in diameter) caused a 1.3% increase in cardiovascular deaths per 5 µg/m^3^ increase in urban polluted air [2]. Combustion of fossil fuels is one of the main generators of PM, including diesel engines. The size of fine diesel exhaust particles (DEP) is normally less than 2.5 µm in diameter, allowing free access to the lungs and subsequently the circulatory system [8]. Furthermore, DEP exposure caused the release of inflammatory mediators associated with blood coagulation and an increase in intracellular calcium levels [9].

The role of the mitochondria in exacerbation of heart disease in diabetics has become increasingly clear [10]. Mitochondria of cardiomyocytes are primary targets for diabetes-related heart disease, suggesting that diabetic cardiomyopathy is a common complication leading to an increased risk for heart failure [1], [10]. Consequences of diabetic cardiomyopathy include systolic dysfunction [11], ventricular hypertrophy [12], hypertension [13], and cardiac contractile protein glycosylation [14]. Furthermore, mitochondria from diabetic hearts have morphological and functional damage coupled with lipid oxidation [10], suggesting an important role for ROS generation in diabetes [15]. These adverse effects of ROS can be reduced by cotreatment with antioxidants [15]. Mitochondria are one of the main ROS generators via single electron leakage to oxygen under various pathophysiological conditions [16], [17], [18], [19]. Since ROS play an important role in various disease states [20], [21], [22], [23], [24], particularly in the development/progression of cardiovascular dysfunction [23], [25], it is very likely that diabetes may induce ROS formation associated with mitochondrial dysfunction in heart cells.

Recent studies of PM exposure have shown that the mitochondria are not involved in particle-induced cellular ROS formation [26]. NADPH oxidase is known to be a source of ROS production in CVDs [27], [28]. The cellular membrane bound NADPH oxidase initiates a single electron transfer to molecular oxygen, resulting in the formation of ROS [27], [29]. Since there is a large distribution of NADPH oxidases located within the cell, it is likely that this enzyme could be another important ROS generator, particularly in the heart [27]. Since previous studies have focused on whole body/organ exposure rather than the direct effects of DEP on ventricular cardiomyocytes, the aim of the present study was to delineate the role of ROS generation in DEP-induced cardiomyocyte dysfunction under diabetes-like conditions. One plausible explanation is that DEP and high glucose alter the local chemical, mechanical, and/or electrical environment through the activation of ROS generators, potentially including the mitochondria and NADPH oxidases.

## Materials and Methods

Adult male Sprague-Dawley rats (250–350 g; Harlan Sprague-Dawley) were maintained on food and water *ad libitum* and all experiments were approved and performed in accordance with the guidelines of the Institutional Laboratory Animal Care and Use Committee at the Research Institute at Nationwide Children's Hospital (IACUC#: AR08-00004).

### Cardiomyocyte isolation

A method similar to previous publications was used [30], [31], [32], with minor modifications. In brief, rats were anesthetized with an injection of pentobarbital sodium (50 mg/kg, i.p.) and heparinized with 0.1 ml of 1000 IU/kg. Following hemithoractomy, the hearts were removed, the aorta was cannulated, and the coronaries were perfused retrogradely at a constant flow rate (4 ml/min) for 5 min with standard Krebs perfusion buffer (in mM: 113 NaCl, 4.7 KCl, 0.6 KH_2_PO_4_, 0.6 Na_2_HPO_4_, 1.2 MgSO_4_-7H_2_O, 12 NaHCO_3_, 10 KHCO_3_, 10 HEPES, 30 Taurine, 0.032 Phenol Red, pH 7.4). The heart was positioned inside a heated dual-walled glass chamber maintained at a constant temperature of 37°C. Ventricular digestion occurred through the addition of calcium, liberase (Roche) and trypsin (GIBCO) into the perfusate, which produced final concentrations of 12.5 µM, 4.5 mg/ml and 0.14 mg/ml, respectively. Following 20 min of enzymatic digestion, the heart was rapidly removed from the perfusion apparatus and minced in 50 ml Krebs standard buffer with 10% fetal bovine serum (SAFC Biosciences) and 12.5 µM CaCl_2_. The mixture was triturated using a sterile pipette and filtered through a sterile cell strainer (BD Falcon, 100 µm in diameter). The filtered solution was titrated in sequence with 150 µl of 10 mM CaCl_2_, 300 µL of 10 mM CaCl_2_, 90 µL of 100 mM CaCl_2_, and 150 µl of 100 mM CaCl_2_ (4 min intervals between each CaCl_2_ addition), and the mixture was centrifuged for 1.5 min at 500 RPM. The supernatant was removed and the pellet dispersed in the plating medium on laminin-coated dishes (Invitrogen). The media contained Minimum Essential Medium with Hanks' salts and L-glutamine (MEM, GIBCO/Invitrogen), 5% bovine calf serum (BCS, Hyclone), 10 mM 2,3-butanedione monoxime (BDM, Sigma), 100 U/ml penicillin (Sigma), 1.8 mM CaCl_2_ and 2 mM L-glutamine (GIBCO/Invitrogen). After 60 min at 37°C in the incubator, the plating media was replaced with fresh culture media that included MEM, 0.1 mg/ml myocyte bovine serum albumin (Sigma), 100 U/ml penicillin and 2 mM L-glutamine (GIBCO/Invitrogen).

### Treatments

DEP (Diesel Particulate Matter, 1.0 µg/ml, National Institute of Standard and Technology, USA) was dissolved by thorough sonication in the contracting buffer (CB). For function studies, isolated ventricular myocytes were divided into eight groups. Control (Ctrl): cells were cultured overnight in standard medium; HG: cells were cultured overnight in a media with a high concentration of glucose (25.5 mM [31], [33]) (i.e. diabetic-like media); DEP: cells were cultured overnight with DEP (0.1 µg/ml); HG+DEP: cells were cultured overnight in the presence of both HG (25.5 mM) and DEP (0.1 µg/ml); The next four groups were similar to the initial four groups except that all were treated with antioxidants (AOX), which included Tiron (4,5-dihydroxy-1,3-benzene disulfonic acid) (0.25 mM) and N-Acetyl-L-cysteine (NAC, 0.5 mM). For confocal studies, myocytes were divided into 17 groups. The first eight groups were all treated overnight as listed above. The next nine groups were also treated overnight, but an additional 1 hr treatment was performed prior to confocal measurement, including: Ctrl+MI (One hr treatment with mitochondria inhibitors including rotenone (1 µg/ml) and TTFA (50 µM)), HG+MI, DEP+MI, HG+DEP+MI, Ctrl+Apo (One hr treatment with apocynin (100 µM)), HG+Apo, DEP+Apo, HG+DEP+Apo, and HG+DEP+MI+Apo. Groups of cells were assessed using the IonOptix video imaging system for real-time contractility, whereas laser scan confocal microscopy (Zeiss LSM510, Carl Zeiss, Jena, Germany) was used for ROS assessment. Live cells were rod-shaped myocytes with distinct sarcomeric edges. In our preparation, cell viability was greater than 75%.

### Cardiomyocyte function

Myocytes were plated in glass-bottom inserts (Cell Micro Controls Co., USA) that were held on a flow chamber on the stage of an inverted Olympus IX-71 microscope. The cells were observed using a 40× objective and superfused with contractile buffer (CB) at 1 ml/min at ∼37°C through a Warner in-line heater associated with an automatic temperature controller (Warner Instrument Co.). The cells were field-stimulated at 1 Hz with a 3 ms duration using the Myopacer Field-Stimulator System (IonOptix, MA) in an apparatus containing two platinum wires on both ends of the insert. The constituents of CB were (in mM, pH 7.4): 131 NaCl, 4 KCl, 10 HEPES, 1 CaCl_2_, 1 MgCl_2_ and 10 glucose. We used the Sarclen Sarcomere Length Acquisition Module (IonOptix) to assess functional properties of the cells. With the IonOptix video imaging system, sarcomere length was recorded using a Myocam-S Digital CCD camera. The following parameters were recorded: sarcomere peak shortening normalized to baseline length (PS, the maximal % change of the sarcomere length from the resting state), sarcomere time-to-90% shortening (TPS_90_, time to 90% of cell contraction), time-to-90% relengthening (TR_90_, time to 90% of cell relaxation), sarcomere departure velocity (+dL/dt, the maximal velocity of cell shortening), and sarcomere return velocity (−dL/dt, the maximal velocity of cell relengthening).

### Intracellular ROS production in cardiomyocytes

The hydroethidine (DHE; dihydroethidium)/ethidium (ET) fluorescence probe was used to measure intracellular ROS formation in cardiomyocytes as described previously [20], [22]. DHE (Invitrogen-Molecular Probes) is highly sensitive to several ROS including superoxide, hydroxyl radical, and peroxynitrite [20], [22], [34]. DHE stock was made in dimethyl sulfoxide (DMSO, Sigma). In the presence of ROS, DHE is instantly dehydrogenated, resulting in the formation of 2-hydroxyethidium (OH-ET). In cells, OH-ET quickly converts to a more stabilized product called ET [35], [36]. ET is a charged molecule and can be trapped intracellularly and thus was used as a valid marker to monitor ROS generation [20], [34], [37], [38]. The DHE assay is widely used for detecting ROS in both cells and intact tissues [39], [40]. Myocytes were loaded with 5 µM HE for 60 min at 37°C. The setup parameters for confocal imaging detection of ROS were illustrated as the following: laser, HeNe; optical slice, <6.6 µm; pinhole, 7.15 air units; scaling range, 0.45×0.45 µm^2^; pixel time, 1.60 µs; objective, Plan-Apochromat 20×0.75; ET excitation, 543 nm; ET emission, and LP 560 nm. The emitted signal captured by a PMT is presented as an image of 1024×1024 pixels on a computer monitor. The imaging data were analyzed using LSM 510 software. All of the fields were randomly selected containing at least 10 cells within each view area. The mean fluorescence intensity of each cell was calculated, and the total cell emission signals per field were averaged for data analyses.

### Statistical Analyses

Values are expressed as mean ± SEM. Data were analyzed using SAS JMP (SAS Institute, Cary, NC) with JMP post hoc analysis, which is a valid technique that is based on comparing obtained differences between two means (treated vs. non-treated). If the difference obtained was greater than or equal to the critical difference, it was considered significant. Each “rat” per day was treated as a random variable with drug treatment in a SAS statistical model. JMP post hoc contrasts were applied to determine differences in mean values at each specific data point if the ANOVA was determined to be significant. p<0.05 was considered statistically significant.

## Results

### Function of cardiomyocytes exposed to HG, DEP, and HG+DEP

**Figure 1:**
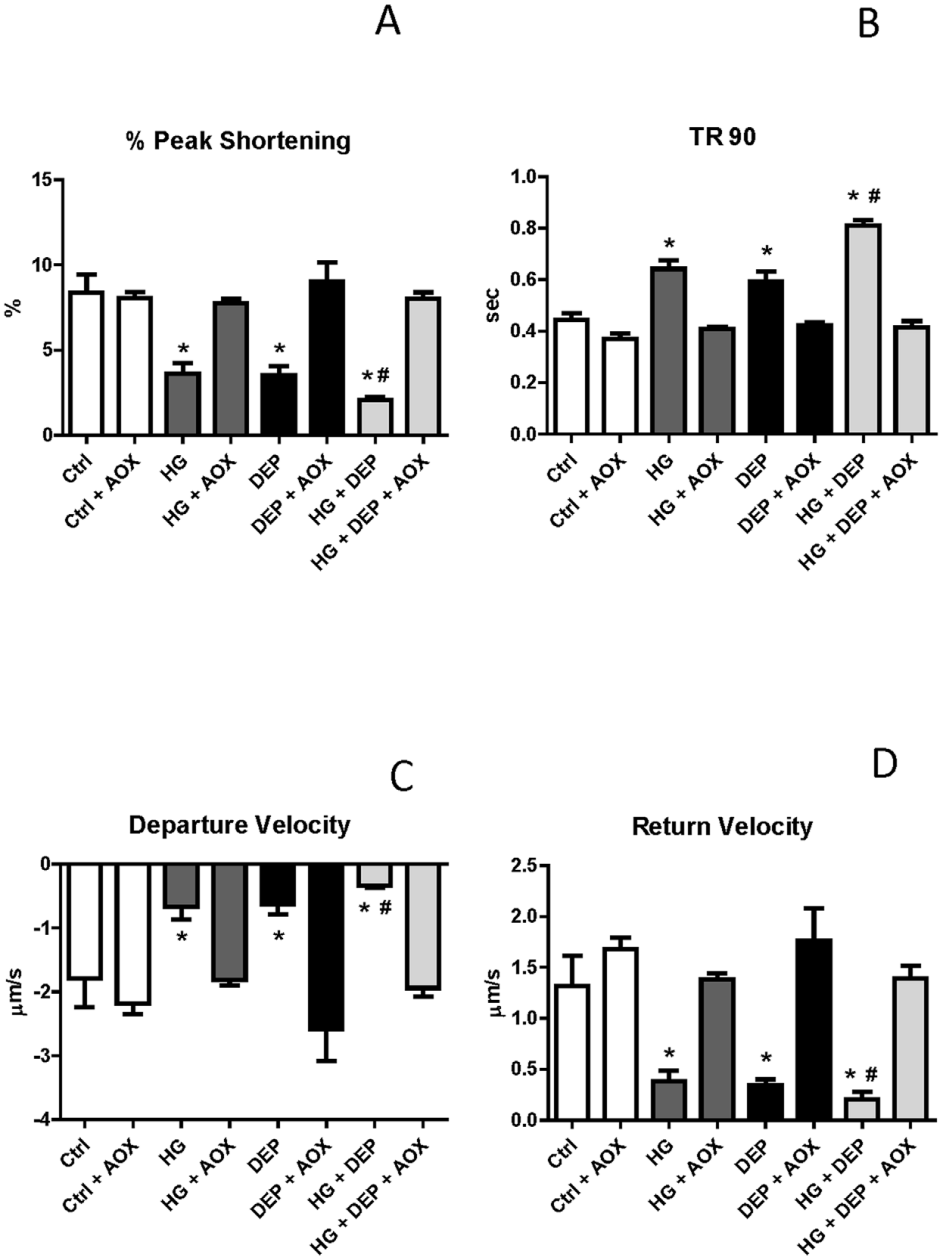
Shortening and relengthening contractility in isolated ventricular myocytes stimulated at 1 Hz. Peak shortening (PS) (A) (% cell length), time-to-90% relengthening (TR_90_) (B) departure (C) and (D) return velocity (±dL/dt µm/s) in control (culture medium overnight; Ctrl), high glucose treatment (25.5 mM overnight; HG), diesel exhaust particle (DEP) treatment (0.1 µg/ml overnight; DEP), HG and DEP treatment (25.5 mM and 0.1 µg/ml overnight); Ctrl with antioxidants (Tiron at 0.25 mM and NAC at 0.5 mM; (AOX)), HG with AOX, DEP with AOX, HG+DEP with AOX. Myocytes were field stimulated with 1 Hz at 37°C. Sample size ranged from 38 to 72 cells per group from 5–8 rats. (*) Significantly compared to Ctrl; (#) significantly compared to HG and DEP (P<0.05).

As shown in Figure 1, overnight DEP exposure caused a significant decrease in peak shortening normalized to baseline PS (Fig. 1A) and a significant increase in TR_90_ (Fig. 1B). Furthermore, sarcomere return velocity +dL/dt (Fig. 1C), and departure velocity −dL/dt (1D) were decreased in DEP exposed cells. Overnight HG treatment also increased TR_90_ (Fig. 1B), but decreased PS (Fig. 1A) and ±dL/dt (Fig. 1C, D). In our model, HG+DEP exposure resulted in the largest increase in TR_90_ (Fig. 1B), and the largest decreases in PS (Fig. 1A) and ±dL/dt (Fig. 1C, D). However, all of these effects were completely abolished when cells were pretreated with a combination of the antioxidants Tiron plus NAC in culture (Fig. 1A–D). All data were obtained at 1 Hz and averaged from 38 to 72 cells from 5–8 rats per group (*p<0.05).

### Cell morphology of isolated ventricular myocytes exposed to DEP

**Figure 2:**
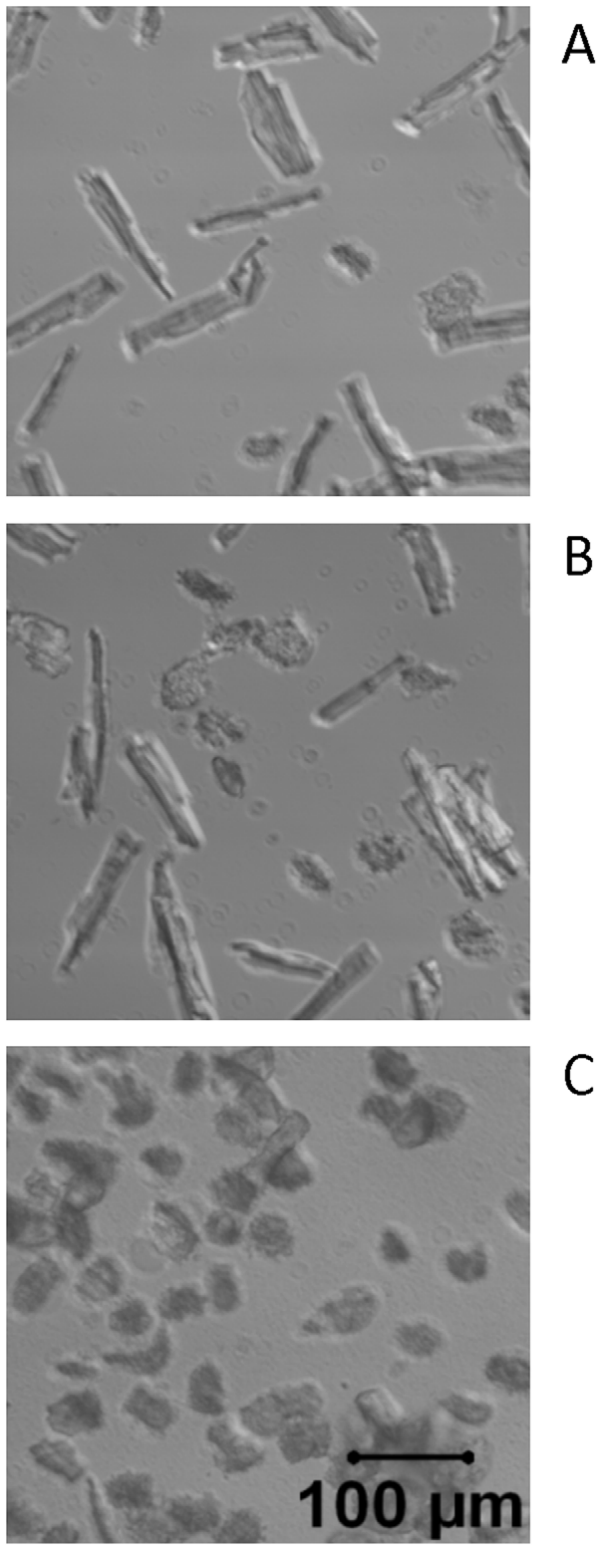
Cell morphology of isolated ventricular myocytes (A–C). Typical myocytes treated overnight with 0 µg/ml (A); 0.1 µg/ml DEP (B); or 1.0 µg/ml DEP (C).

**Figure 3:**
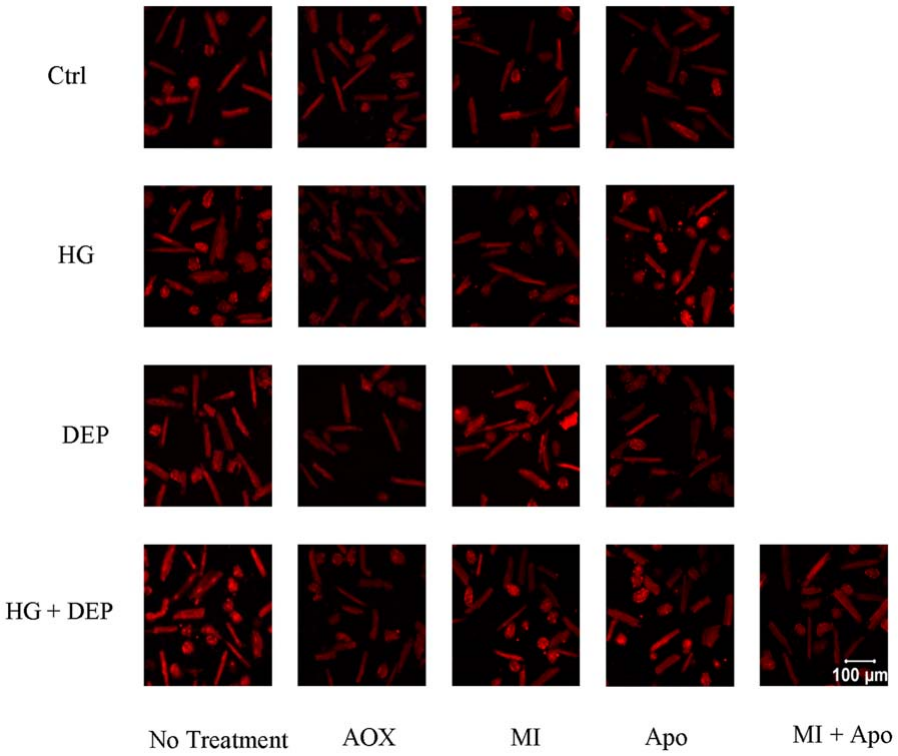
Intracellular reactive oxygen species (ROS) generation in isolated ventricular myocytes. Typical ET confocal emission images of Ctrl *versus* Ctrl with AOX (Tiron at 0.25 mM and NAC at 0.5 mM), Ctrl with MI (rotenone at 1 µg/ml and the mitochondrial complex II inhibitor (MI) thenoyltrifluoroacetone (TTFA) at 50 µM), and Ctrl with apocynin (100 µM, Apo); Typical ET emission images treated with HG (25.5 mM) *versus* HG with AOX, HG with MI, and HG with Apo; Typical ET images of DEP (0.1 µg/ml) *versus* DEP with AOX, DEP with MI, and DEP with Apo; Typical ET images of HG (25.5 mM) and DEP (0.1 µg/ml) *versus* HG and DEP with AOX, HG and DEP with MI, HG+DEP with Apo, and HG+DEP with MI and Apo.

**Figure 4:**
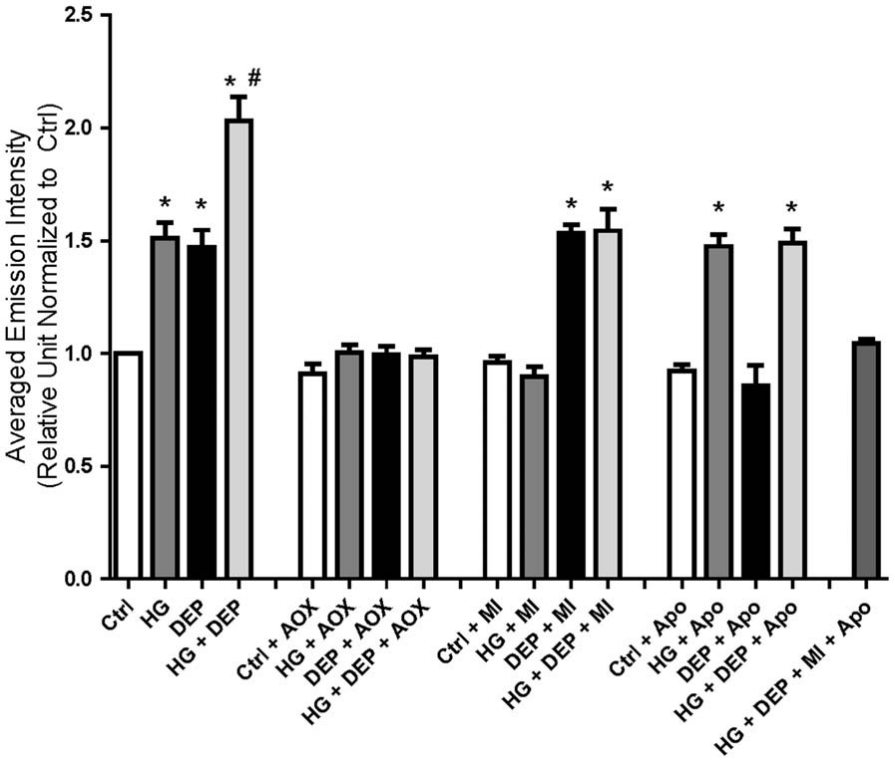
Summary data for intracellular ROS generation as shown by changes in ET fluorescence in isolated ventricular myocytes. Sample size ranged from 100 to 360 cells from 5–8 rats. (*) Significantly different compared to Ctrl, HG+AOX, HG+MI, DEP+AOX, DEP+MI, HG+DEP+MI+Apo; (#) Significantly different compared to HG and DEP (P<0.05).

In Figure 2A, control myocytes are shown without DEP overnight treatment. 1.0 µg/ml DEP overnight exposure (Fig. 2C) caused massive cell death compared to the control group (Fig. 2A). 0.1 µg/ml DEP caused significant ROS generation (Fig. 2B) and cardiomyocyte dysfunction with minimal effects on cell morphology (Figs. 1, 2, 3, 4). Therefore, we utilized 0.1 µg/ml DEP as the effective treatment dose throughout the rest of our experiments.

### Intracellular ROS production in myocytes exposed to overnight HG, DEP, HG+DEP

Figure 3 is comprised of ET images of loaded myocytes, suggesting significant increases in ROS generation with either HG or DEP exposure overnight, that was highest with combined treatment of HG+DEP overnight. Antioxidant (Tiron and NAC, AOX) co-culture abolished these signals in all treatment groups. Mitochondrial blockers (rotenone and TTFA, MI) blocked ET emission in overnight HG-treated cells, while the NADPH oxidase blocker, apocynin (Apo) blocked ET fluorescence in overnight DEP-treated cells. Both MI and Apo partially blocked ET emission in cells exposed to HG+DEP overnight. However, MI+Apo completely blocked the ET signal compared to control (Figure 4). In addition, antioxidants used in this study had no artificial effects on control cells (Figs. 3 and 4).

## Discussion

Air pollution exposure and diabetes mellitus can both cause cardiovascular dysfunction [1], [2], [3]. The present study is the first to show that DEP exposure exacerbates contractile dysfunction in isolated myocytes exposed to high glucose (HG) media *in vitro*, which can be blocked by antioxidant co-culture. We have also shown that ROS generation in cardiomyocytes exposed to HG could potentially involve the mitochondria, while ROS generation induced by DEP is likely mediated through NADPH oxidase-dependent pathways. Furthermore, in HG-treated myocytes, we found that overnight exposure to diesel particulate matter (DEP) exacerbates the already increased TR_90_ and intracellular ROS, with the largest effects on cardiomyocyte function (evidenced by changes in PS and ±dL/dt) in our experimental settings. The levels of DEP (0.1 µg/ml) used in this study were maintained within a physiological range compared to higher doses previously used [41], [42]. Furthermore, inhibition of both NADPH oxidase and the mitochondria resulted in a relatively weaker ROS signal compared to those in the HG+DEP treated group, suggesting complex mechanisms involving multiple ROS generators. Our data provide molecular insight into the mechanisms of diabetic cardiomyopathy in highly polluted urban areas [2], [3].

### DEP exacerbates HG-induced cardiomyocyte dysfunction

Previous studies [2], [4], [5] have shown that long-term exposure to PM potentiates cardiovascular disease progression. Our current study further explored the effect of direct exposure of DEP to cardiomyocytes treated with HG in culture (simulated *in vitro* diabetes), since diabetes is another major risk factor for CVD development [1], [43]. 25.5 mM glucose in culture is commonly accepted as a standard diabetic-like media [30], [33], [44]. As part of our examination of cardiomyocyte contractility, either HG or DEP exposure significantly reduced myocyte function (Figure 1). The largest functional decreases were observed in HG+DEP treated cells (i.e. simulating the diabetic environment). Previous studies have shown that ROS play an active role in both diabetes [15] and DEP-related disease progression [45], [46]. Mitochondria, one of the main ROS generators in cardiomyocytes, are also subject to effects of diabetes [1], [10]. Findings in the present study suggest that both HG and DEP exposure involve oxidative stress-generating pathways.

### ROS production in isolated cardiomyocytes treated with HG+DEP

ROS generation is a key mechanism in the generation of both CVD and PM-induced disease [10], [15], [45], [46], [47], [48]. Figure 3 suggests that the mechanisms whereby HG or DEP decrease cardiac function may involve an unknown oxidative pathway. As illustrated in Figure 1, functional properties of ventricular myocytes treated with HG, DEP or HG+DEP were completely restored with the co-culture of the antioxidants Tiron and NAC [45], [48], suggesting that ROS play a critical role in these processes. Although the specific mechanisms involved remain unclear, these results are consistent with previous studies showing that ROS generation can inhibit cardiac muscle function through various pathways, including oxidative modification of sarcoplasmic reticulum Ca^2+^ ATPase and contractile elements involved in excitation-contraction coupling [20], [21]. We further confirmed intracellular ROS production using laser scan confocal fluorescent microscopy. ROS formation was observed in HG, DEP and HG+DEP treated cardiomyocytes (Figs. 3 and 4). We have found that ROS generation was also blocked by co-culture of antioxidants (Figs. 3 and 4). Tiron and NAC produced no artificial effects on basal fluorescence level or myocyte function in control cells (Figs. 1, 3 and 4). Our findings suggest that ROS formation is involved in both HG and DEP-treated myocytes.

### Molecular sources of ROS production in cardiomyocytes treated with DEP

A number of molecular sources for ROS in muscle have been proposed. The mitochondria are possible ROS generators via electron leakage to molecular oxygen under pathological conditions [16], [17], [49]. Our findings (Figs. 3 and 4) are consistent with previous studies of mitochondria isolated from diabetic hearts; in these studies, the authors observed morphological and functional damage to the mitochondria in diabetic samples, presumably caused by ROS generation [10], [15]. In our model, mitochondria complex I and II blockers were used to test whether the mitochondria play a role in HG-treated cells [50]. We found that these inhibitors completely diminished intracellular ROS generation, suggesting that the mitochondria are possible ROS generators in isolated myocytes exposed to HG media. Furthermore, contractility was completely recovered following exposure to antioxidants, as shown in Figure 1, confirming that ROS play a role in cardiomyocyte dysfunction induced by HG or DEP.

Although mitochondria are regarded as possible sources of ROS in diabetic models, Mo et al. [26] have recently shown that mitochondria are not directly involved in PM-induced cellular ROS formation. Following activation, membrane bound NADPH oxidase initiates single-electron transfers to molecular oxygen, resulting in the formation of ROS [33], [51], [52]. Of particular importance is the fact that NADPH oxidase is largely distributed within heart cells [27]. Mo et al. [26] also illustrated that NADPH oxidase appears to be the source of ROS generation following *in vitro* DEP treatment in pulmonary microvascular endothelial cells, highly consistent with our current results (Figs. 3 and 4). In the present study, we have shown that DEP-induced ROS generation was completely diminished in apocynin-treated cells (Figs. 3 and 4). Therefore, considering previous studies [26], [53], (Figs. 1, 3 and 4), it is likely that DEP induces NADPH oxidase to generate ROS in our model.

### Conclusions and future directions

This study demonstrated that ROS are produced following DEP and/or HG exposure to isolated cardiomyocytes. Antioxidant co-culture completely abolished the adverse effects of HG or DEP on the functional properties of isolated myocytes. However, the mechanisms whereby HG activates mitochondrial ROS generation or DEP activates NADPH oxidase-dependent ROS generation are still under investigation. It is likely that HG and/or DEP could activate mitochondria or NADPH oxidase via uncertain signaling pathways such as cytokines (TNF-α, IL-6) and/or protein kinases [15], [29]. Additional studies will be performed to determine the physiological level of particles within the bloodstream of animals in a highly polluted environment. It is worth noting that mitochondrial blockers have no effect on ROS generation in DEP-treated cells, while apocynin exerts no effect on ROS generation in cells exposed to HG media (Figs. 3 and 4). Therefore, we conclude that DEP exacerbates myocardial dysfunction in isolated cardiomyocytes exposed to HG media, and that the cardiotoxicity of HG or DEP are mediated through distinct and disparate ROS generation pathways.
